# Diaqua­bis(2,2′-biimidazole)zinc(II) 4,4′-di­carboxybiphenyl-3,3′-di­carboxyl­ate

**DOI:** 10.1107/S1600536809008022

**Published:** 2009-03-28

**Authors:** Jie Kang, Chang-Cang Huang, Zhi-Qing Jiang, Sheng Huang, Shuang-Lu Huang

**Affiliations:** a College of Pharmacy, Fujian Medical University, Fuzhou, Fujian 350004, People’s Republic of China; b State Key Laboratory Breeding Base of Photocatalysis, Fuzhou University, Fuzhou 350002, People’s Republic of China

## Abstract

In the title compound, [Zn(C_6_H_6_N_4_)_2_(H_2_O)_2_](C_16_H_8_O_8_), the Zn^II^ atom, located on an inversion centre, is coordinated by two aqua and two bidentate biimidizole ligands, resulting in a slightly distorted octa­hedral ZnO_2_N_4_ geometry. The four N atoms from the two biimidizole ligands lie in the equatorial plane and the two aqua O atoms lie in the axial sites. The biphenyl­tetra­carboxyl­ate anion also lies on an inversion centre. The Zn^II^ complex cation and the anion are held together by N—H⋯O hydrogen bonds, forming a zigzag chain along [2
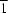
1]. The chains are further connected by water mol­ecules *via* O—H⋯O hydrogen bonds.

## Related literature

For general background, see: Hagrman *et al.* (1999 ▶); Jia *et al.* (2007 ▶); Kortz *et al.* (2003 ▶).
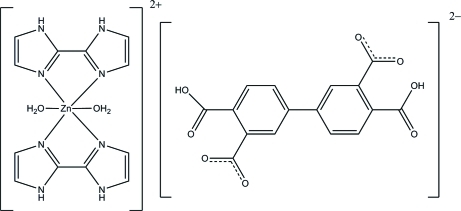


## Experimental

### 

#### Crystal data

[Zn(C_6_H_6_N_4_)_2_(H_2_O)_2_](C_16_H_8_O_8_)
*M* *_r_* = 697.92
Triclinic, 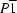

*a* = 8.2133 (16) Å
*b* = 9.810 (2) Å
*c* = 10.498 (2) Å
α = 63.72 (3)°
β = 68.00 (3)°
γ = 83.85 (3)°
*V* = 701.4 (2) Å^3^
*Z* = 1
Mo *K*α radiation
μ = 0.95 mm^−1^
*T* = 293 K
0.12 × 0.10 × 0.08 mm

#### Data collection

Bruker APEXII CCD diffractometer
Absorption correction: multi-scan (**SADABS**; Bruker, 2001 ▶) *T*_min_ = 0.894, *T*_max_ = 0.928
5074 measured reflections
2674 independent reflections
2579 reflections with *I* > 2σ(*I*)
*R*_int_ = 0.022

#### Refinement

*R*[*F* ^2^ > 2σ(*F* ^2^)] = 0.036
*wR*(*F* ^2^) = 0.096
*S* = 1.00
2674 reflections
218 parameters
1 restraint
H atoms treated by a mixture of independent and constrained refinement
Δρ_max_ = 0.28 e Å^−3^
Δρ_min_ = −0.22 e Å^−3^

### 

Data collection: *APEX2* (Bruker, 2004 ▶); cell refinement: *SAINT-Plus* (Bruker, 2001 ▶); data reduction: *SAINT-Plus*; program(s) used to solve structure: *SHELXS97* (Sheldrick, 2008 ▶); program(s) used to refine structure: *SHELXL97* (Sheldrick, 2008 ▶); molecular graphics: *SHELXTL* (Sheldrick, 2008 ▶); software used to prepare material for publication: *SHELXTL*.

## Supplementary Material

Crystal structure: contains datablocks global, I. DOI: 10.1107/S1600536809008022/is2385sup1.cif

Structure factors: contains datablocks I. DOI: 10.1107/S1600536809008022/is2385Isup2.hkl

Additional supplementary materials:  crystallographic information; 3D view; checkCIF report

## Figures and Tables

**Table 1:** Selected bond lengths (Å)

Zn1—O1*W*	2.135 (2)
Zn1—N3	2.1419 (18)
Zn1—N2	2.1625 (19)

**Table 2:** Hydrogen-bond geometry (Å, °)

*D*—H⋯*A*	*D*—H	H⋯*A*	*D*⋯*A*	*D*—H⋯*A*
N1—H1*A*⋯O1^i^	0.88	1.94	2.802 (3)	169
N4—H4*A*⋯O2^i^	0.90	1.89	2.791 (3)	176
O1*W*—H1*W*⋯O4^ii^	0.81	1.90	2.683 (2)	162
O1*W*—H2*W*⋯O2^iii^	0.79	1.98	2.751 (3)	164
O3—H3*A*⋯O1	0.93 (3)	1.52 (3)	2.434 (3)	165 (4)

Symmetry codes: (i) 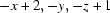; (ii) 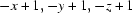; (iii) 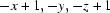.

**Fig. 1.**
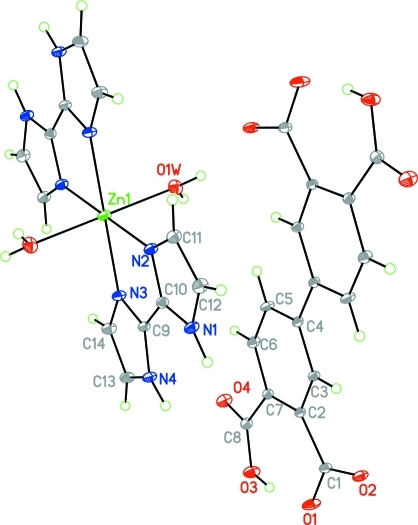
The molecular components of the title compound, drawn with 30% probability displacement ellipsoids for the non-hydrogen atoms.

**Fig. 2.**
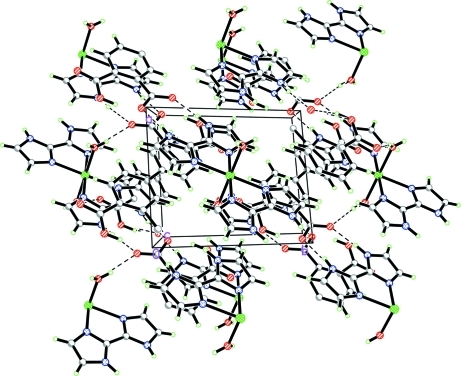
A packing diagram of the title compound formed with the hydrogen bonds (dashed lines).

## supplementary crystallographic information

### Comment

Design and construction of metal-organic frameworks (MOFs) have attracted considerable attention in recent years, not only for their intriguing structural motifs but also for their potential applications in the areas of catalysis, separation, gas absorption, molecular recognition, nonlinear optics and magnetochemistry (Hagrman *et al.*, 1999; Jia *et al.*, 2007; Kortz *et al.*, 2003). In this paper, we report the structure of the title compound, (I).

As shown in Fig. 1, the Zn^II^ atom (site symmetry 1) is bonded to two aqua and two bidentate biimidizole ligands, to result in a slightly distorted octahedral ZnO_2_N_4_ geometry for the central metal. The Zn^II^ atom lies on an inversion centre, as a consequence which the asymmetric unit comprises a half of the molecule. The four nitrogen atoms belonging to two biimidizole ligands lie in the equatorial plane and the two aqua oxygen atoms lie in the axial coordination sites. The bonds around Zn is listed in Table 1. The 3,3',4,4'-biphenyl tetracarboxylate acts as negative electron balance. With two kinds of hydrogen bonds of N4—H4A···O2 and N1—H1A···O1, a zigzag chain is formed. Furthermore, a 3-D frameworks is constructed with O1W—H2W···O2 and O1W—H1W···O4 along the *c* axis, shown in Fig. 2.

### Experimental

All chemicals and Teflon-lined stainless steel autoclave were purchased from Jinan Henghua Sci. & Tec. Co. Ltd. A mixture of 3,3',4,4'-biphenyl tetracarboxylic acid (0.1 mmol), zinc(II) sulfate (0.1 mmol), and diimdazole (0.1 mmol) in 10 ml distilled water sealed in a 25 ml Teflon-lined stainless steel autoclave was kept at 433 K for three days. Colorless crystals suitable for X-ray were obtained.

### Refinement

Atom H3A on O3 was located in a difference Fourier map and refined with an O—H distance [0.93 (1) Å] restraint. O-bound H atoms except H3A and N-bound H atoms were located in a difference Fourier map and were constrained as riding, with *U*_iso_(H) = 1.2*U*_eq_(O or N). Other H atoms were placed in calculated positions (C—H = 0.93 Å) and refined as riding, with *U*_iso_(H) = 1.2*U*_eq_(C).

### Crystal data

**Table:**

[Zn(C_6_H_6_N_4_)_2_(H_2_O)_2_](C_16_H_8_O_8_)	*Z* = 1
*M**_r_* = 697.92	*F*(000) = 358
Triclinic, *P*1	*D*_x_ = 1.652 Mg m^−^^3^
Hall symbol: -P 1	Mo *K*α radiation, λ = 0.71073 Å
*a* = 8.2133 (16) Å	Cell parameters from 2674 reflections
*b* = 9.810 (2) Å	θ = 3.4–26.0°
*c* = 10.498 (2) Å	µ = 0.95 mm^−^^1^
α = 63.72 (3)°	*T* = 293 K
β = 68.00 (3)°	Block, colorless
γ = 83.85 (3)°	0.12 × 0.10 × 0.08 mm
*V* = 701.4 (2) Å^3^	

### Data collection

**Table:**

Bruker APEXII CCD diffractometer	2674 independent reflections
Radiation source: fine-focus sealed tube	2579 reflections with *I* > 2σ(*I*)
graphite	*R*_int_ = 0.022
φ and ω scans	θ_max_ = 26.0°, θ_min_ = 3.4°
Absorption correction: multi-scan (*SADABS*; Bruker, 2001)	*h* = −9→10
*T*_min_ = 0.894, *T*_max_ = 0.928	*k* = −12→12
5074 measured reflections	*l* = −12→11

### Refinement

**Table:**

Refinement on *F*^2^	Primary atom site location: structure-invariant direct methods
Least-squares matrix: full	Secondary atom site location: difference Fourier map
*R*[*F*^2^ > 2σ(*F*^2^)] = 0.036	Hydrogen site location: inferred from neighbouring sites
*wR*(*F*^2^) = 0.096	H atoms treated by a mixture of independent and constrained refinement
*S* = 1.00	*w* = 1/[σ^2^(*F*_o_^2^) + (0.052*P*)^2^ + 0.4235*P*] where *P* = (*F*_o_^2^ + 2*F*_c_^2^)/3
2674 reflections	(Δ/σ)_max_ = 0.018
218 parameters	Δρ_max_ = 0.28 e Å^−^^3^
1 restraint	Δρ_min_ = −0.22 e Å^−^^3^

### Special details

**Table:**

Geometry. All e.s.d.'s (except the e.s.d. in the dihedral angle between two l.s. planes) are estimated using the full covariance matrix. The cell e.s.d.'s are taken into account individually in the estimation of e.s.d.'s in distances, angles and torsion angles; correlations between e.s.d.'s in cell parameters are only used when they are defined by crystal symmetry. An approximate (isotropic) treatment of cell e.s.d.'s is used for estimating e.s.d.'s involving l.s. planes.
Refinement. Refinement of *F*^2^ against ALL reflections. The weighted *R*-factor *wR* and goodness of fit *S* are based on *F*^2^, conventional *R*-factors *R* are based on *F*, with *F* set to zero for negative *F*^2^. The threshold expression of *F*^2^ > σ(*F*^2^) is used only for calculating *R*-factors(gt) *etc*. and is not relevant to the choice of reflections for refinement. *R*-factors based on *F*^2^ are statistically about twice as large as those based on *F*, and *R*- factors based on ALL data will be even larger.

### Fractional atomic coordinates and isotropic or equivalent isotropic displacement parameters (Å^2^)

**Table:**

	*x*	*y*	*z*	*U*_iso_*/*U*_eq_	
Zn1	0.5000	0.5000	0.0000	0.03706 (14)	
C1	0.8917 (3)	−0.0165 (3)	0.6897 (3)	0.0359 (5)	
C2	0.7565 (3)	0.0726 (2)	0.6220 (2)	0.0299 (4)	
C3	0.6742 (3)	−0.0082 (2)	0.5790 (2)	0.0317 (4)	
H3	0.7113	−0.1040	0.5887	0.038*	
C4	0.5400 (3)	0.0464 (2)	0.5224 (2)	0.0300 (4)	
C5	0.4858 (3)	0.1890 (3)	0.5114 (3)	0.0399 (5)	
H5	0.3941	0.2288	0.4771	0.048*	
C6	0.5668 (3)	0.2719 (2)	0.5509 (3)	0.0392 (5)	
H6	0.5280	0.3673	0.5415	0.047*	
C7	0.7037 (3)	0.2197 (2)	0.6041 (2)	0.0305 (4)	
C8	0.7779 (3)	0.3350 (3)	0.6322 (3)	0.0375 (5)	
C9	0.7614 (3)	0.3778 (2)	0.1372 (2)	0.0328 (4)	
C10	0.7227 (3)	0.2594 (2)	0.1042 (2)	0.0336 (5)	
C11	0.5985 (3)	0.1497 (3)	0.0235 (3)	0.0436 (6)	
H19	0.5263	0.1307	−0.0179	0.052*	
C12	0.7149 (4)	0.0538 (3)	0.0759 (3)	0.0473 (6)	
H20	0.7368	−0.0416	0.0771	0.057*	
C13	0.8671 (3)	0.5175 (3)	0.2050 (3)	0.0440 (6)	
H21	0.9329	0.5524	0.2414	0.053*	
C14	0.7403 (3)	0.5901 (3)	0.1520 (3)	0.0436 (6)	
H22	0.7041	0.6853	0.1456	0.052*	
N1	0.7933 (3)	0.1248 (2)	0.1265 (2)	0.0406 (4)	
H1A	0.8659	0.0821	0.1742	0.049*	
N2	0.6044 (3)	0.2788 (2)	0.0414 (2)	0.0366 (4)	
N3	0.6732 (2)	0.5025 (2)	0.1091 (2)	0.0372 (4)	
N4	0.8794 (3)	0.3831 (2)	0.1946 (2)	0.0402 (4)	
H4A	0.9538	0.3110	0.2211	0.048*	
O1	0.9901 (3)	0.0489 (2)	0.7164 (3)	0.0592 (5)	
O2	0.9025 (2)	−0.15120 (19)	0.7137 (2)	0.0528 (5)	
O3	0.9163 (3)	0.3100 (2)	0.6670 (3)	0.0576 (5)	
O4	0.7063 (3)	0.4532 (2)	0.6186 (2)	0.0536 (5)	
O1W	0.2826 (2)	0.4101 (2)	0.2126 (2)	0.0517 (5)	
H1W	0.2680	0.4378	0.2782	0.078*	
H2W	0.2367	0.3277	0.2477	0.078*	
H3A	0.962 (5)	0.217 (2)	0.673 (5)	0.100 (13)*	

### Atomic displacement parameters (Å^2^)

**Table:**

	*U*^11^	*U*^22^	*U*^33^	*U*^12^	*U*^13^	*U*^23^
Zn1	0.0416 (2)	0.0294 (2)	0.0549 (3)	0.00961 (15)	−0.03317 (18)	−0.01971 (17)
C1	0.0359 (11)	0.0351 (11)	0.0469 (12)	0.0071 (9)	−0.0249 (10)	−0.0197 (10)
C2	0.0321 (10)	0.0270 (10)	0.0351 (10)	0.0038 (8)	−0.0184 (9)	−0.0129 (8)
C3	0.0346 (11)	0.0260 (10)	0.0424 (11)	0.0073 (8)	−0.0229 (9)	−0.0155 (9)
C4	0.0355 (11)	0.0254 (10)	0.0351 (10)	0.0042 (8)	−0.0201 (9)	−0.0130 (8)
C5	0.0489 (13)	0.0323 (11)	0.0604 (14)	0.0156 (10)	−0.0411 (12)	−0.0242 (11)
C6	0.0502 (13)	0.0280 (11)	0.0542 (13)	0.0122 (10)	−0.0320 (11)	−0.0220 (10)
C7	0.0349 (11)	0.0284 (10)	0.0343 (10)	0.0029 (8)	−0.0174 (9)	−0.0152 (8)
C8	0.0452 (13)	0.0319 (11)	0.0446 (12)	0.0026 (9)	−0.0226 (10)	−0.0195 (10)
C9	0.0317 (10)	0.0327 (11)	0.0402 (11)	0.0070 (8)	−0.0200 (9)	−0.0166 (9)
C10	0.0349 (11)	0.0291 (10)	0.0396 (11)	0.0059 (8)	−0.0182 (9)	−0.0145 (9)
C11	0.0566 (15)	0.0321 (11)	0.0559 (14)	0.0036 (10)	−0.0337 (12)	−0.0201 (11)
C12	0.0608 (16)	0.0309 (12)	0.0636 (16)	0.0106 (11)	−0.0330 (13)	−0.0253 (11)
C13	0.0460 (13)	0.0461 (14)	0.0579 (15)	0.0050 (11)	−0.0316 (12)	−0.0281 (12)
C14	0.0490 (14)	0.0380 (12)	0.0640 (15)	0.0105 (10)	−0.0332 (12)	−0.0308 (12)
N1	0.0456 (11)	0.0326 (10)	0.0545 (12)	0.0137 (8)	−0.0324 (10)	−0.0192 (9)
N2	0.0418 (10)	0.0300 (9)	0.0489 (11)	0.0083 (8)	−0.0282 (9)	−0.0181 (8)
N3	0.0394 (10)	0.0347 (10)	0.0532 (11)	0.0105 (8)	−0.0302 (9)	−0.0233 (9)
N4	0.0394 (10)	0.0393 (10)	0.0552 (12)	0.0115 (8)	−0.0315 (9)	−0.0223 (9)
O1	0.0659 (12)	0.0518 (11)	0.1051 (16)	0.0251 (9)	−0.0670 (12)	−0.0479 (11)
O2	0.0539 (11)	0.0330 (9)	0.0920 (14)	0.0141 (8)	−0.0539 (11)	−0.0248 (9)
O3	0.0620 (12)	0.0441 (10)	0.1003 (15)	0.0128 (9)	−0.0549 (12)	−0.0412 (11)
O4	0.0706 (12)	0.0399 (10)	0.0807 (13)	0.0161 (9)	−0.0472 (11)	−0.0387 (9)
O1W	0.0637 (12)	0.0427 (9)	0.0564 (11)	−0.0089 (8)	−0.0204 (9)	−0.0268 (8)

### Geometric parameters (Å, °)

**Table:**

Zn1—O1W	2.135 (2)	C8—O3	1.288 (3)
Zn1—O1W^i^	2.135 (2)	C9—N3	1.326 (3)
Zn1—N3^i^	2.1419 (18)	C9—N4	1.334 (3)
Zn1—N3	2.1419 (18)	C9—C10	1.445 (3)
Zn1—N2^i^	2.1625 (19)	C10—N2	1.321 (3)
Zn1—N2	2.1625 (19)	C10—N1	1.341 (3)
C1—O2	1.231 (3)	C11—C12	1.358 (4)
C1—O1	1.258 (3)	C11—N2	1.368 (3)
C1—C2	1.528 (3)	C11—H19	0.9300
C2—C3	1.395 (3)	C12—N1	1.364 (3)
C2—C7	1.411 (3)	C12—H20	0.9300
C3—C4	1.392 (3)	C13—C14	1.351 (4)
C3—H3	0.9300	C13—N4	1.361 (3)
C4—C5	1.390 (3)	C13—H21	0.9300
C4—C4^ii^	1.490 (4)	C14—N3	1.370 (3)
C5—C6	1.376 (3)	C14—H22	0.9300
C5—H5	0.9300	N1—H1A	0.8755
C6—C7	1.391 (3)	N4—H4A	0.9008
C6—H6	0.9300	O3—H3A	0.93 (3)
C7—C8	1.522 (3)	O1W—H1W	0.8119
C8—O4	1.217 (3)	O1W—H2W	0.7930
			
O1W—Zn1—O1W^i^	180.00 (10)	O4—C8—C7	119.0 (2)
O1W—Zn1—N3^i^	88.19 (8)	O3—C8—C7	120.8 (2)
O1W^i^—Zn1—N3^i^	91.81 (8)	N3—C9—N4	111.41 (19)
O1W—Zn1—N3	91.81 (8)	N3—C9—C10	119.57 (19)
O1W^i^—Zn1—N3	88.19 (8)	N4—C9—C10	129.0 (2)
N3^i^—Zn1—N3	180.0	N2—C10—N1	111.27 (19)
O1W—Zn1—N2^i^	87.51 (8)	N2—C10—C9	119.67 (19)
O1W^i^—Zn1—N2^i^	92.49 (8)	N1—C10—C9	129.1 (2)
N3^i^—Zn1—N2^i^	79.56 (7)	C12—C11—N2	109.2 (2)
N3—Zn1—N2^i^	100.44 (7)	C12—C11—H19	125.4
O1W—Zn1—N2	92.49 (8)	N2—C11—H19	125.4
O1W^i^—Zn1—N2	87.51 (8)	C11—C12—N1	106.6 (2)
N3^i^—Zn1—N2	100.44 (7)	C11—C12—H20	126.7
N3—Zn1—N2	79.56 (7)	N1—C12—H20	126.7
N2^i^—Zn1—N2	180.0	C14—C13—N4	106.3 (2)
O2—C1—O1	122.0 (2)	C14—C13—H21	126.8
O2—C1—C2	117.98 (19)	N4—C13—H21	126.8
O1—C1—C2	120.0 (2)	C13—C14—N3	109.8 (2)
C3—C2—C7	118.34 (19)	C13—C14—H22	125.1
C3—C2—C1	113.56 (18)	N3—C14—H22	125.1
C7—C2—C1	128.06 (18)	C10—N1—C12	107.05 (19)
C4—C3—C2	123.86 (19)	C10—N1—H1A	128.5
C4—C3—H3	118.1	C12—N1—H1A	124.1
C2—C3—H3	118.1	C10—N2—C11	105.86 (19)
C5—C4—C3	116.66 (19)	C10—N2—Zn1	110.26 (14)
C5—C4—C4^ii^	122.8 (2)	C11—N2—Zn1	143.84 (16)
C3—C4—C4^ii^	120.6 (2)	C9—N3—C14	105.05 (18)
C6—C5—C4	120.6 (2)	C9—N3—Zn1	110.75 (14)
C6—C5—H5	119.7	C14—N3—Zn1	143.92 (16)
C4—C5—H5	119.7	C9—N4—C13	107.4 (2)
C5—C6—C7	123.1 (2)	C9—N4—H4A	126.2
C5—C6—H6	118.5	C13—N4—H4A	126.4
C7—C6—H6	118.5	C8—O3—H3A	113 (3)
C6—C7—C2	117.43 (18)	Zn1—O1W—H1W	121.7
C6—C7—C8	113.31 (18)	Zn1—O1W—H2W	121.9
C2—C7—C8	129.25 (19)	H1W—O1W—H2W	111.7
O4—C8—O3	120.1 (2)		

Symmetry codes: (i) −*x*+1, −*y*+1, −*z*; (ii) −*x*+1, −*y*, −*z*+1.

### Hydrogen-bond geometry (Å, °)

**Table:**

*D*—H···*A*	*D*—H	H···*A*	*D*···*A*	*D*—H···*A*
N1—H1A···O1^iii^	0.88	1.94	2.802 (3)	169
N4—H4A···O2^iii^	0.90	1.89	2.791 (3)	176
O1W—H1W···O4^iv^	0.81	1.90	2.683 (2)	162
O1W—H2W···O2^ii^	0.79	1.98	2.751 (3)	164
O3—H3A···O1	0.93 (3)	1.52 (3)	2.434 (3)	165 (4)

Symmetry codes: (iii) −*x*+2, −*y*, −*z*+1; (iv) −*x*+1, −*y*+1, −*z*+1; (ii) −*x*+1, −*y*, −*z*+1.
